# Supplementary material for: OxyR regulates the oxidative stress response in Zymomonas mobilis during oxic growth and anoxic biofuel fermentation
Source: Appl Environ Microbiol. 2026 Aug 4;92(9):e01044-26. doi: 10.1128/aem.01044-26 (PMC13599515; doi:10.1128/aem.01044-26)
Supplement: Supplementary file 1 [file aem.01044-26-s0001.docx]

**Supplementary Information**

**Figure S1. Leaky expression of pRL814 and overexpression of pRL814-*oxyR***. **A)** ZM4 and ZM4 pRL814 (containing a GFP gene) were grown anoxically in ZRMG in a 96-well plate for 48 h with no IPTG inducer added. The absolute fluorescence values of GFP were measured at 460/40 nm excitation, 528/20 nm emission. Lines represent the average of 3 biological replicates, and standard error values are displayed with transparent ribbon. ZM4 pRL814 (purple), ZM4 pRL814-*oxyR* (pink)*, ΔoxyR* pRL814 (green), and *ΔoxyR* pRL814-*oxyR* (blue) were grown in anoxic **(B)** and oxic conditions **(C)** for 48 h in ZRMG with 100 µM IPTG. Points represent the average of 3 biological replicates, and error bars represent standard error.

**Figure S2. Differentially expressed genes in ∆*oxyR***. The row-centered z-scores of the Fragments Per Kilobase Million (FPKM) value for each differentially expressed gene (DEG) in this dataset are shown. DEGs were defined as genes that had significant differential expression (> +1 or < -1 log_2_ fold change and False Discovery Rate (Benjamini-Hochberg adjusted p-value, FDR < 0.05) in at least one contrast between conditions. Hierarchal clustering was used. FPKM values were calculated as the average across three or four biological replicates.

**Figure S2. Anoxic growth of *ΔoxyR* in hydrolysate inhibitors.** ZM4 pRL814, *ΔoxyR* pRL814, and *ΔoxyR* pRL814-*oxyR* were grown anaerobically in a BioTek plate reader in ZRMG for 48 hours, with varying concentrations of **A)** acetic acid, **B)** formic acid, **C)** ferulic acid, and **D)** coumaric acid. The concentrations added of each compound were determined by the measured concentration found in 7% switchgrass AFEX hydrolysate, as reported in (2). Lines represent the average of 3 biological replicates, and standard error values are displayed with transparent ribbon.

**Figure S3. Addition of pRL814-*ahpC* does not rescue oxic growth of *∆oxyR***. **A)** ZM4 pRL814 (purple), *ΔoxyR* pRL814 (green), and *ΔoxyR* pRL814-*ahpC (*dark blue) were grown in oxic ZRMG for 100 hours. Points represent the average of 3 biological replicates, and error bars represent standard error. **B)** CellROX results from Figure 2, including the strain *ΔoxyR* pRL814-*ahpC (*dark blue). Strains were grown for 24 hours in oxic ZRMG. The absolute fluorescence values were measured at 480 nm excitation, 520 nm emission after 25 µM CellRox incubation for 30 min. 100 µM menadione was added to ZM4 20 minutes prior to CellROX addition as a positive control for ROS (yellow). No CellRox was added to ZM4 as a negative control (red). Bars represent the average of 3 biological replicates, and error bars represent standard error.

**Figure S4. Anoxic growth of *ΔoxyR* in hydrolysate inhibitors.** ZM4 pRL814, *ΔoxyR* pRL814, and *ΔoxyR* pRL814-*oxyR* were grown anoxically in ZRMG in a 96-well plate for 48 hours, with varying concentrations of **A)** acetic acid, **B)** formic acid, **C)** ferulic acid, and **D)** coumaric acid. The concentrations added of each compound were determined by the measured concentration found in 7% switchgrass AFEX hydrolysate, as reported in (2). Lines represent the average of 3 biological replicates, and standard error values are displayed with transparent ribbon.

**Figure S5. Catalase does not reduce measured peroxide levels in hydrolysate.** The Pierce Peroxide Assay was used to measure peroxide levels in oxic AFEX hydrolysate after 1 h of treatment with catalase enzyme, in concentrations ranging from 0 – 5 U/mL. Absorbance was measured at 595 nm. Points represent the average of 3 technical replicates, and error bars represent standard error.

**Table S1. Differentially expressed transcriptional regulators in *∆oxyR***. The genes that were differentially expressed in *∆oxyR* vs ZM4 encoding for transcriptional regulators are shown below. Their predicted functions are listed if known.

| Gene | Name | Predicted Regulator | Predicted Function |
| --- | --- | --- | --- |
| *ZMO0153* | none | YebC/PmpR family DNA-binding transcriptional regulator | none |
| *ZMO0221* | none | CarD family transcriptional regulator | regulates transcription |
| *ZMO1107* | none | Lrp/AsnC family transcriptional regulator | none |
| *ZMO1202* | *nrdR* | transcriptional regulator NrdR | regulates expression of ribonucleotide reductase enzymes |
| *ZMO1387* | none | response regulator transcription factor | none |
| *ZMO1412* | *mucR* | MucR family transcriptional regulator | global regulator of cell processes including cell cycle, motility, and exopolysaccharide production |
| *ZMO1816* | *nifA* | nif-specific transcriptional activator NifA | regulates expression of nitrogen fixation genes |

**Table S2. Chemical composition of synthetic hydrolysate SynHv4.1**. The source and concentration of each compound is reported below. SynHv4.1 was produced at the Experimental Fermentation Laboratory at the University of Wisconsin-Madison. Adapted from Barten et al (38).

| **Component** | **Source (Catalog # or CAS #,Vendor)** | **Concentration in mM** |
| --- | --- | --- |
| **Major Sugars, Acids and Organics (mM)** | | |
| D-Glucose |  | 333 |
| D-Xylose |  | 253 |
| Sodium formate | S648-500, Fisher Scientific | 8.5 |
| Sodium nitrate | S343-500, Fisher Scientific | 1.1 |
| Sodium succinate.6H_2_O | S413-500, Fisher Scientific | 0.3 |
| Glycerol | G33-500, Fisher Scientific | 2.9 |
| Sodium acetate | S8750-250G, Fisher Scientific | 44.2 |
| D-Mannose | 3458-28-4, Acros | 2.2 |
| L-Arabinose | 28697-53-2, Acros | 5.9 |
| D-Fructose | L96-500, Fisher Scientific | 11.9 |
| D-Galactose | G0750-500G, Sigma Aldrich | 2.2 |
| D-Ribose | 132361000, Acros | 1.3 |
| Acetamide | 00160, Fluka | 53.1 |
| **Abundant Metals and Inorganics (mM)** | | |
| (NH_4_)_2_SO_4_ | 7783-20-2, Acros | 3 |
| NH_4_Cl | A661-500, Fisher Scientific | 24 |
| MgCl_2_·6H_2_O | M33-500, Fisher Scientific | 13.3 |
| CaCl_2_·2H_2_O | C79-500, Fisher Scientific | 1.2 |
| KCl | P217-500, Fisher Scientific | 30.4 |
| NaCl  KH_2_PO_4_ (as a buffer)  K_2_HPO_4_ (as a buffer) | S271-500, Fisher Scientific  P386-500, Fisher Scientific  P288-500, Fisher Scientific | 0.8  4.1  7.7 |
| **Trace Metals, Inorganics, Vitamins and other compounds (μM)** | | |
| ZnCl_2_ | 96469, Fluka | 10 |
| MnCl_2_·4H_2_O | M87-500, Fisher Scientific | 64 |
| CuCl_2_·2H_2_O | 307483-100G, Sigma Aldrich | 1.5 |
| CoCl_2_·6H_2_O | 60820, Fluka | 0.02 |
| H_3_BO_3_ | B6768-500G, Sigma Aldrich | 29.3 |
| (NH_4_)_6_Mo_7_O_24_•4H_2_O | 09878, Fluka | 0.26 |
| Fe (III) citrate·4H_2_O | F6129-250g, Sigma Aldrich | 6.7 |
| Sodium citrate | BP327-500, Fisher Scientific | 1000 |
| Pyridoxine.HCl | BP267750, Fisher Scientific | 2.14 |
| Nicotinic Acid | 1282950000, Acros | 26.78 |
| Biotin | B4501-10G, Sigma Aldrich | 0.1 |
| Myo-Inositol  Thiamine HCl  Calcium Pantothenate  Betaine.H_2_O  Choline Chloride  D/L-Carnitine.HCl | 57570-25G, Fluka  BP892-100, Sigma Aldrich  C8731-25G, Sigma Aldrich  14300, Fluka  67-48-1, Acros  461-05-2, Acros | 56  0.4  3  700  300  300 |
|  |  |  |
| **Amino Acids and Nucleotides (μM)** | | |
| L-Alanine | 05129-25G, Fluka | 381 |
| L-Arginine.HCl | 11039, Sigma Aldrich | 108 |
| L-Asparagine | 11149-25G-F, Sigma Aldrich | 113 |
| D/L-Aspartic acid.K | A2025-100G, Sigma Aldrich | 195 |
| L-Cysteine.HCl | 30129-25G, Sigma Aldrich | 50 |
| L-Glutamine | 49419-25G, Sigma Aldrich | 76 |
| L-Glutamic acid.K  Glycine | G1501-500G, Sigma Aldrich  410225-250G, Sigma Aldrich | 155  296 |
| L-Histidine | 71-00-1, Acros | 16 |
| L-Isoleucine | 58879, Sigma Aldrich | 59 |
| L-Leucine | 61819-25G, Sigma Aldrich | 229 |
| L-Lysine.HCl | L5626-100G, Sigma Aldrich | 79 |
| L-Methionine | 64319-25G-F, Sigma Aldrich | 100 |
| L-Phenylalanine | P5482-25G, Sigma Aldrich | 207 |
| L-Proline | 81709-25G, Sigma Aldrich | 141 |
| L-Serine | 84959-100G, Sigma Aldrich | 144 |
| L-Threonine | 89179-10G, Sigma Aldrich | 100 |
| L-Tryptophan | 93659-10G, Sigma Aldrich | 50 |
| L-Tyrosine | 93829-25G, Sigma Aldrich | 133 |
| L-Valine  Adenine  Cytosine  Uracil  Guanine | 72-18-4, Acros  1152, Calbiochem  71-30-7, Alfa Aesar  6630, Calbiochem  73-40-5, Alfa Aesar | 126  50  50  50  50 |
| **Other Components (g/L)** | | |
| Polysorbate 80 (Tween 80)  Ergosterol | 59924-1KG-F, Fluka  E6510-10G, Sigma | 1  10 |
| **Lignocellulose-Derived**  **Inhibitors (LCDIs)** | **Source (Cat. # or CAS #, Vendor)** | **Concentration in**  **μM** |
| **Amides, Imidazoles and Pyrazines (μM)** |  |  |
| Feruloyl amide | NA | 701 |
| Coumaroyl amide | NA | 1104 |
| 2-Methylimidazole  4(5)-Methylimidazole  2, 4-Dimethylimidazole  2-Methylpyrazine  2, 6-Dimethylpyrazine | M50850-100G, Sigma  199885-50G, Sigma  A11949, Alfa Aesar  W330906-100G-K, Sigma  W327301-50G-K, Sigma | 73  164  7.6  38  14.3 |
| **Acids (μM)** |  |  |
| *p*-Coumaric acid | C9008-25G, Sigma | 146 |
| Ferulic acid | W518301-100G, Sigma | 33 |
| Benzoic acid | 242381-100G, Sigma | 344 |
| Vanillic acid | H36001-25G, Sigma | 134 |
| 4-hydroxybenzoic acid | 240141-50G, Sigma | 62 |
|  |  |  |
| **Alcohols, Aldehydes and Ketones (μM)** |  |  |
| Vanillin | V1104-100G, Sigma | 84 |
| 4-Hydroxybenzeldehyde | S7602-25G, Sigma | 70 |
| Coniferyl alcohol | 223735-1G, Sigma | 55 |
| 4-Hydroxyacetophenone | 278564-100G, Sigma | 33 |
| Furfural | F94-500, Fisher | 47 |
